# The Influence of Genetics in Myopia Control: A Pilot Study

**DOI:** 10.3390/jcm10040808

**Published:** 2021-02-17

**Authors:** Cristina Alvarez-Peregrina, Miguel Ángel Sánchez-Tena, Clara Martinez-Perez, Catalina Santiago-Dorrego, Thomas Yvert, Cristina Andreu-Vazquez, Cesar Villa-Collar

**Affiliations:** 1 Department of Pharmacy, Biotechnology, Nutrition, Optics and Optometry, Faculty of Biomedical and Health Science, Universidad Europea de Madrid, 28670 Madrid, Spain; cristina.alvarez@universidadeuropea.es (C.A.-P.); miguelangel.sanchez@universidadeuropea.es (M.Á.S.-T.); villacollarc@gmail.com (C.V.-C.); 2 Department of Physiotherapy, Faculty of Sport Sciences, Universidad Europea de Madrid, 28670 Madrid, Spain; catalina.santiago@universidadeuropea.es (C.S.-D.); thomaspaul.yvert@universidadeuropea.es (T.Y.); 3 Department of Medicine. Faculty of Biomedical and Health Science, Universidad Europea de Madrid, 28670 Madrid, Spain; cristina.andreu@universidadeuropea.es

**Keywords:** myopia, genetics, childhood

## Abstract

Background: Many epidemiological and experimental studies have established that myopia is caused by a complex interaction between common genetic and environmental factors. The objective of this study was to describe and compare the allelic and genotypic frequencies of the rs524952 (*GJD2*), rs8000973 (*ZIC2*), rs1881492 (*CHRNG*), rs1656404 (*PRSS56*), rs235770 (*BMP2*), and rs7744813 (*KCNQ5*) SNPs (single-nucleotide polymorphism) between responder and nonresponder patients who had undergone a two-year treatment with lenses for myopia control. Method: Twenty-eight participants from the MiSight Assessment Study Spain (MASS), who had received treatment for myopia control for two years with MiSight contact lenses, were examined. The criteria for better/worse treatment response was the change in the axial length (< / ≥ 0.22 mm two years after the treatment). The clinical procedure consisted of the extraction of a saliva sample, and the participants also underwent an optometric examination. Genetic data were analyzed using SNPStats software (Catalan Institute of Oncology, Barcelona, Spain), and statistical analysis was performed using SPSS v.25 (SPSS Inc., Chicago, IL, USA). Demographic variables were analyzed using the Student’s t-test. Results: The T allele, the one with the lowest frequency, of the “rs235770” SNP was associated with a better treatment response [AL/CR (axial length/corneal radius): OR = 3.37; CI = 1.079–10.886; SE (spherical equivalent): OR = 1.26; CI: = 0.519–57.169; *p* = 0.019). By performing haplotype analysis, significant differences were found between the rs235770…rs1881492 and rs235770–rs1656404 polymorphisms. The latter presented a strong linkage disequilibrium with each other (r^2^ ≥ 0.54). Conclusion: The result of lens therapies for myopia control could vary depending on genetic variants. Studies with a larger sample are needed to confirm the results presented in this pilot study.

## 1. Introduction

Myopia is the most common refractive error globally. It is considered a public health problem that results in significant visual loss and is associated with a wide variety of ocular pathologies [1].

Nowadays, pathological myopia is one of the leading causes of visual impairment in Asian and Occidental populations. As the prevalence of myopia and pathological myopia increases throughout the world, the need for the active prevention of myopia progression and the treatment of its potential complications increases [2].

Many epidemiological and experimental studies have analyzed the role of environmental and genetic factors in the development of myopia. Environmental risk factors can only explain a limited proportion of the overall variance, whereas the importance of genetic factors in the susceptibility to myopia is widely known [3]. In particular, since the appearance of the first genome-wide association studies (GWAS) in 2009, many common genetic variants that are associated with myopia and refractive error have been successfully identified [4].

As a result, in the last years, some consortia such as the International Consortium for Refractive Error and Myopia (CREAM), the 23andMe Research Team, and the UK Biobank Eye and Vision Consortium have tried to identify the genetic variants that are associated with different refractive errors, and in particular with myopia. In 2013, 39 SNPs (single-nucleotide polymorphism) associated with myopia were found [5,6]. In 2016, Tideman et al. [7], as members of the CREAM consortium, analyzed the influence of these SNPs on axial length (AL) and corneal radius (CR) depending on age. In those younger than 10 years old, three loci (*GJD2, CHRNG, ZIC2*) were linked with AL/CR (axial length/corneal radius). In the age group between 10 and 25 years old, there were four linked loci (*BMP2, KCNQ5, A2BP1, CACNA1D*). In adults, there were 20 linked loci. In 2018, using a meta-analysis that included 160,420 subjects, the number of genetic polymorphisms linked with refractive errors increased from 39 to 161 [8]. In the large-scale study published in 2020 in Nature Genetics, Hysi et al. [9] conducted a meta-analysis of GWAS, which involved 542,934 European participants, in which 336 new genetic loci, linked with the refractive error, were identified.

As such, all of these studies provide new potential knowledge regarding the evolution of myopia and perhaps promising leads for future therapies. However, the genetic architecture and its molecular mechanisms have not been made clear, and genetic risk prediction models are still improving. So, through a better understanding of the genetic influence, we will be able to establish foundations for the relationship between heredity and the appearance of myopia. Likewise, it will be possible to improve the efficacy of current treatment methods for myopia control (optical, pharmacological, environmental, and surgical). In 2019, Wildsoet et al. [10] concluded that the efficacy of these methods varies significantly depending on the individual and that no treatment is considered to be 100% effective in all patients. Therefore, more research is needed to understand the factors and mechanisms underlying the said variability.

Consequently, based on the study by Tideman et al. [7], the six SNPs with the highest association with the development of myopia have been selected. So, the objective of this study was to describe and compare the allelic and genotypic frequencies of the rs524952, rs8000973, rs1881492, rs1656404, rs235770, and rs7744813 SNPs of the *GJD2, ZIC2, CHRNG, PRSS56, BMP2*, and *KCNQ5* genes, respectively, between responder and nonresponder patients who had undergone a two-year treatment with lenses for myopia control. Besides, associations between each SNP and several endophenotypes (spherical equivalent (SE), AL, and CR) were analyzed.

## 2. Experimental Section

### 2.1. Study Design and Approval of the Ethics Committee

A pilot, analytical, longitudinal, ambispective, and case-control study was performed. The study was positively evaluated by the CEIM–Regional Ethics Committee of the Department of Health of the Community in Madrid. Besides, the study was developed following the standards recognized by the Declaration of Helsinki by the World Medical Association (64th General Meeting, Fortaleza, Brazil, October 2013), as well as the Good Clinical Practice Guidelines, and it was also in line with the Spanish legislation for the treatment of samples of human origin (Law 14/2007) on biomedical research and the Royal Decree 1716/2011.

Likewise, all of the participants declared a clear understanding of the study objectives by signing the informed consent form.

### 2.2. Selection of SNPs

A total of six SNPs were included in this analysis. The SNPs were selected based on the study of Tideman et al. [7]. Tideman et al. included 18 cohorts from 8 different countries in Europe, Asia, and Oceania with 5490 children under the age of 10, 5000 subjects between the ages of 10 and 25, and 16,274 adults over the age of 25. The results showed an association with the AL/CR ratio in 9 of the 39 SNPs in children under 10 years old, remaining in 3 SNPs after Bonferroni correction; 10 SNPs remaining in 5 in the group aged between 10 and 25; and 31 SNPs that remained in 19 in the adult group.

Table 1 shows the characteristics of the nine SNPs associated with the AL/CR ratio.

Considering the best values in the significance of the beta effect, the SNPs described in *GJD2, ZIC2, PRSS56, BMP2*, and *KCNQ5* were chosen for this study.

### 2.3. Study Population and Inclusion Criteria

All the participants in the MiSight Assessment Study Spain (MASS) who had worn Misight (CooperVision, Pleasanton, CA, USA) for two years to control myopia, fitting in the Novovisión Ophthalmological Clinic and Universidad Europea de Madrid, between September 2013 and June 2016 (age at the start of treatment: 8–12 years, myopia level at the beginning of the treatment: −0.75 to –4.00 D) [11] were invited to participate in this study. So, 28 of the 41 participants were finally enrolled in this new study.

Following the MASS study, the value of 0.22 mm change in axial length was chosen as cut-off point to select the subjects in responders and nonresponders. Therefore, patients were classified according to their response to treatment as responders (axial length below 0.22 mm two years after the treatment) and nonresponders (patients with an axial length equal to or greater than 0.22 mm two years after treatment).

Furthermore, the MASS study showed that the samples were homogeneous in demographic, refractive, binocular, accommodative, and biometric baseline data.

### 2.4. Clinical Procedure

The protocol was divided into two parts: the extraction of a saliva sample and the performance of an optometric examination:

(a) Oral mucosa sample extraction: Genomic DNA was extracted from oral mucosa samples collected with a sterile swab using the commercial High Pure PCR Template Preparation Kit (Roche) once the treatment had finished. The extracted samples were kept in the UEM Biomedicine laboratory at −20 °C for analysis. The genetic variants were analyzed using a PCR-Q in Step One Plus equipment (Life Technologies, Foster City, CA, USA) with TaqMan probes predesigned by Life Technologies. The DNA samples were kept by the Biomedicine unit of the Universidad Europea de Madrid until the end of the study. Once the study concluded, the DNA samples were destroyed unless the patient or guardian had granted authorization, through the informed consent form, for the research team to incorporate his/her sample into a collection of biological samples that were registered in the Carlos III Institute of Health for subsequent studies within this line of research.

(b) Optometric examination: The standard procedure consisted of taking visual acuity without and with correction, objective refraction using the retinoscopy technique without cycloplegia, subjective refraction, accommodative and binocular tests (cover–uncover, alternating cover test, ocular motility, accommodative delay, amplitude of accommodation, and near point of convergence), ocular biometry (axial length) using the IOLMaster^®^ (5.4.4.006; Carl Zeiss Jen GmbH, Jena, Germany), corneal topography (Wavelight Allegro Topolyzer, TX, USA). Finally, the front segment (eyelid, eyelashes, lid margin, cornea, conjunctiva, and crystalline) was evaluated using a slit lamp.

### 2.5. Statistical Analysis

The allelic and genotypic frequencies, as well as the Hardy–Weinberg equilibrium (HWE), linkage disequilibrium (LD), and odds ratio (OR), were analyzed with the SNPStats software (Catalan Institute of Oncology, Barcelona, Spain; http://bioinfo.iconcologia.net/SNPstats) [12] and the Haploview 4.1 software (Broad Institute, Cambridge, MA, USA) [13].

Statistical analysis was performed using the SPSS 25.0 program (SPSS Inc., Chicago, IL, USA). For the descriptive analysis, the absolute (*n*) and relative (%) frequencies were used to express the qualitative variables and the mean ± standard deviation (SD) (or the median and interquartile range, IQR) for the quantitative ones as a function of its parametric behavior. For this purpose, the Shapiro–Wilk test of normality was used.

The AL/CR relation was calculated by dividing the average AL of the right and left eye (in mm) by the average CR of the right and left eye (in mm). The value of the spherical equivalent (SE) and AL/CR used was that obtained after two years of treatment.

Differences between groups were analyzed by Chi-square test (qualitative variables) and Student’s t-test (quantitative variables). Levene’s test was performed to check the homogeneity between the variances, with the result that the variances were homogeneous (*p* > 0.05).

The OR value was used to analyze the association of multiple SNPs with the endophenotypes of myopia between the groups (responders and nonresponders) and the regressions between quantitative (SE, CR, and AL) variables were used to show the relationships. To evaluate the statistical significance, a cleavage site *p* > 0.05 was considered. The p values and the OR in the genotype models were adjusted for age and sex.

To analyze the differences in the genotype and allelic frequencies of the polymorphisms between responders and nonresponders, these were calculated based on the three heredity models: additive (R/R vs. R/nR vs. nR/nR), dominant (R/R + R/nR vs. nR/nR), and recessive (R/R vs. R/nR + nR/nR), where R was the risk allele and nR was the no-risk allele.

## 3. Results

### 3.1. Clinical and Demographic Characteristics

A total of 28 subjects aged between 14 and 20 years (mean age: 17.25 ± 1.48) participated in this study; 60.7% (*n* = 17) of the participants were women and 39.3% were men (*n* = 11).

With regards to gender, the mean value of the SE (RE+LE/2) in male participants was −2.42 ± 1.32D (range: −0.87D–−5.00D) and in female participants it was −3.80 ± 1.50D (range: −1D–−7.37D). The mean AL value in male participants was 24.60 ± 0.55 mm (range:23.56–25.43 mm) and in female participants, it was 25.01 ± 0.76 mm (range: 23.25–26.32 mm). Significant differences were found between gender and the SE value (*p* = 0.019; CI: 0.24–2.50); however, no significant differences were found between gender and AL (*p* > 0.05; CI: −0.96–0.13).

With regards to age, the value of SE (14 years: −3.12 ± 0.18D; 20 years: −5.03 ± 1.29D) and AL (14 years: 24.94 ± 0.28 mm; 20 years: 25.21 ± 1.25 mm) increased progressively over the years (*p* < 0.05).

Table 2 (responders: *n* = 15; nonresponders: *n* = 13) shows the clinical characteristics of the participants based on their response to treatment. Of all of the responder participants, 63.3% had low myopia (−0.5D < SE > −3D) and 36.7% had moderate myopia (−3D < SE > −6D). Of all of the nonresponders, 19.2% had low myopia (−0.5D < SE > −3D), 65.4% had moderate myopia (−3D < SE > −6D), and 15.4% had high myopia (SE > −6D). SE mean value was higher in responders than nonresponders (−2.62 ± 1.35 vs. −3.98 ± 1.50, respectively, *p* = 0.019; 95% CI of the difference: −2.46–0.24).

### 3.2. Allele and Genotypic Frequencies

The observed genotype frequencies for these six SNPs were all in HWE for the responder and nonresponder groups (*p* > 0.05).

#### 3.2.1. AL/CR

Table 3 shows the results of the allelic association for the six SNPs (adjusted for age, gender, and AL/CR). The comparison of allele frequencies between the responder and nonresponder groups revealed a nominally significant difference for the rs235770 polymorphism (*p* = 0.049). In contrast, none of the five remaining SNPs showed a significant association with the axial length and keratometry (*p* > 0.05). Table 4 shows the results of the genotype association of the six SNPs, which were calculated for each of the three heredity models (additive, dominant, and recessive). There was no significant association for any of the six SNPs in the additive or dominant models. Furthermore, the rs235770 SNP showed significant differences between the responders and nonresponders in the recessive heredity model (*p* = 0.043).

#### 3.2.2. SE

Table 5 shows the results of the allelic and genotypic association for the six SNPs (adjusted for age, gender, and SE). The comparison of allele frequencies between the responder and nonresponder groups revealed a nominally significant difference for the rs235770 (*p* = 0.009). On the contrary, none of the five remaining SNPs showed a significant association with the SE (*p* > 0.05).

Table 6 shows the results of the genotype association of the six SNPs, which has been calculated for each of the three heredity models (additive, dominant, and recessive). There was no significant association between responders and nonresponders in the three heredity models of the “rs235770” SNP.

### 3.3. Haplotype Analysis

**Figure 1:**
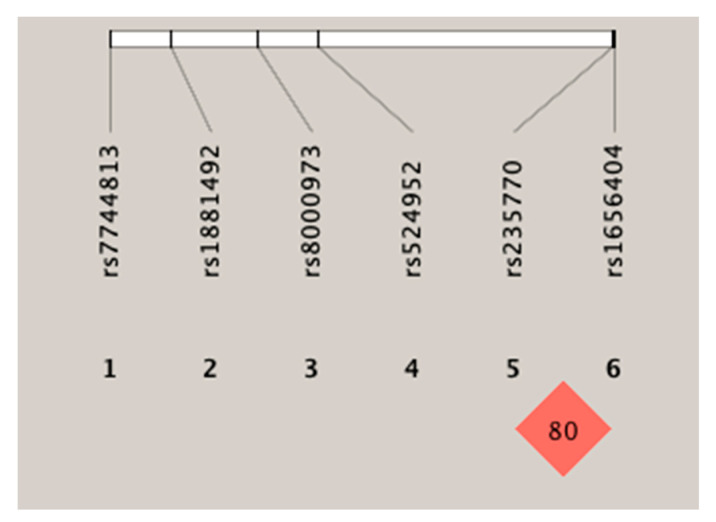
Linkage disequilibrium of the six SNPs (single-nucleotide polymorphism).

Haplotype analysis was performed to help understand the effects of the rs235770 polymorphism of the BMP2 gene on the manifestation of response to treatment. Figure 1 shows the linkage disequilibrium patterns for the six SNPs. These six SNPs were not located in a haplotype block. However, two SNPs (rs235770 and rs1656404) had a strong linkage disequilibrium with each other (r^2^ ≥ 0.54).

Table 7 shows the linkage disequilibrium analysis for the six polymorphisms. A significant association was found between the rs235770–rs1881492 and rs235770–rs1656404 polymorphisms.

Also, the haplotype analysis was performed with the most significant result from the linkage disequilibrium analysis. The most significant difference was observed in the association of the rs235770–rs1656404 polymorphisms (*p* = 0.0286). The GT haplotype was strongly associated with response to treatment (*p* = 0.008). The GT haplotype was more frequent in responders than in nonresponders (50% compared to 16.53%), suggesting a possible effect. In turn, an association was found for the AC haplotype, although it was not significant and this was present in 13.3% of the responders compared to just 5% of the nonresponders (Table 8). The GT and AC haplotype subjects had an OR of 22.99 and 5.06, respectively. That is to say, a greater risk of response to treatment.

## 4. Discussion

This study provides new evidence about how genetic variants influence the results of myopia control therapy with MiSight. Despite the considerable number of studies on myopia control, no treatment is 100% effective. This is probably due to the limited knowledge of the etiology of the complex and multifactorial condition that is myopia. As a result, most optical and pharmacological treatments are based on different causal theories [14]. For this reason, we designed this pilot study in which the rs524952, rs8000973, rs1881492, rs1656404, rs235770, and rs7744813 SNPs of the *GJD2, ZIC2, CHRNG, PRSS56, BMP2*, and *KCNQ5* genes, respectively, were analyzed and compared between responder and nonresponder patients who had undergone a two-year treatment with lenses for myopia control. According to many GWAS, some genes involved along the retina-to-sclera signaling cascade have been discovered. Genetic changes in individual loci only cause small changes in the phenotype, but collectively these disturbances are responsible for more significant changes in the retina-to-sclera signaling cascade, therefore explaining the differences in the refractive error between one individual and another [6,15,16]. The changes in the synthesis of retinoic acid (RA) of the retina and choroid, as well as the RA effects on scleral growth, suggest that RA plays an important role in ocular growth regulation and, consequently, in the development of myopia. It seems to be part of the retina-to-sclera signaling cascade and possibly the effector of scleral extracellular change [17,18,19].

As such, in the first large-scale GWAS, which was conducted by members of the CREAM consortium, the inferred tracts included neurotransmission (*GRIA4*), ion transportation (*KCNQ5*), retinoic acid metabolism (*RDH5*), extracellular matrix remodeling (*LAMA2, BMP2*), and ocular development (*SIX6, PRSS56*) [6,15]. In another GWAS performed by the 23andMe consortium, a set of overlapped tracts was identified: neuronal development (*KCNMA1, RBFOX1, LRRC4C, NGL-1, DLG2, TJP2*), extracellular matrix remodeling (*ANTXR2, LAMA2*), visual cycle (*RDH5, RGR, KCNQ5*), corporal and ocular growth (*PRSS56, BMP4, ZBTB38, DLX1*), and retinal ganglion cells (*ZIC2, SFRP1*) [17]. The recent study by Tedja et al. [8] confirmed the previous findings and identified the functional contributions in the development of refractive errors in all of the cell types of the neurosensory retina: the retinal pigment epithelium, the vascular endothelium, and the extracellular matrix. Furthermore, novel mechanisms such as rod-and-cone bipolar synaptic neurotransmission, anterior segment morphology, and angiogenesis were present in the newly identified genes. Therefore, they concluded that refractive errors are caused by a light-dependent retina-to-sclera signaling cascade.

The present study has unveiled a possible association between rs235770 polymorphism in the *BMP2* gene and the response to treatment that needs to be confirmed in a new study with a larger sample. In turn, it has been found that the T allele, the one with the lowest frequency, could present a greater risk of response to treatment. This result is in line with those recorded in the study by Tideman et al. [7], in which the risk allele of the *BMP2* gene was associated with a lower AL/CR ratio in the group of children aged 10 years and lower. The study conducted by Li et al. [20] found that the *BMP2* gene may be involved in the development of myopia, but it does not have a primary role in the retinal and choroidal signals regulating scleral remodeling. Curiously, *BMP2* gene expression studies performed on chickens showed that the mRNA of this gene in the retinal pigment epithelium presents a positive or negative regulation depending on the dynamic image. This means that when the image is focused behind the retina, the mRNA is regulated negatively and the vitreous chamber is enlarged, therefore suggesting that the *BMP2* gene plays a bidirectional role in modulating ocular growth and that the *BMP2* gene could be used in therapeutic interventions for controlling myopia [21].

The *BMP2* gene is one of the most widely studied growth factors in the BMP family and it is essential for the development of the retina, meaning, therefore, that it plays important roles in embryogenesis and osteogenesis [22]. Furthermore, BMP signaling is neuroprotective for retina ganglion cells after damage and it is involved in glial cell proliferation [23,24]. As such, *BMP2* can act as a negative growth regulator in the retina and RPE. The study that was conducted by Mathura et al. [25] observed a decreased level of *BMP2* in the retina during the development of myopia, but this level increased after recovery from myopia. In this way, as this alteration occurred following significant structural change, the retinal level of *BMP2* is likely associated with ocular growth and the development of myopia.

Nonetheless, the effect of *BMP2* alleles on controlling myopia is still unknown and to be able to explore its underlying mechanisms, further research will be required.

Besides, this study did not find an association between the rs524952 (*GJD2* gene), rs8000973 (*ZIC2* gene), rs1881492 (*CHRNG* gene), rs1656404 (*PRSS56* gene), and rs7744813 (*KCNQ5* gene) polymorphisms and the response to the myopia control treatment. The studies by Simpson et al. [26] and Verhoeven et al. [5] found that these genes were involved in the development of myopia.

Through haplotype analysis, an association between the rs1656404 and rs235770 polymorphisms was found. In this sense, the study by Paylaki et al. [27] identified the PRSS56 gene as a potential therapeutic target for modulating ocular growth aimed at preventing or slowing down myopia. This suggests a possible relationship between the PRSS56 gene and *BMP2*, although there is still no scientific evidence.

In turn, a significant association was observed for the GT haplotype, as this was present approximately two times more often in the responder group than in the nonresponder group. Therefore, the rs235770 T allele affected the response for the myopia control treatment. However, according to the study conducted by Yoshikawa et al. [28], the strength of association of a single SNP only reflects signals that include nearby SNPs with moderate LD and it is far from reflecting the genetic influences of the gene itself.

Consequently, a significant association was found in this study between the SNP “rs235770” with AL/CR. Nevertheless, and despite having obtained a significant *p* value (<0.05), it is not possible to confirm whether an association with SE exists, as the confidence interval crosses 1. The authors believe that this is due to the small size of the sample as well as to the CI calculations, which are usually very conservative, especially in the cases of small samples and exact estimates.

It should be noted, SNPs that originate in genes affect the gene product, but do not modify the protein product of genes. In this way, whether this change contributes to a disease phenotype is dependent on the specific consequence of the particular genetic variant and disease type [29].

One of the limitations of this study is the low sample size. Limited sample sizes can sometimes lead to false positive or false negative results in an association study. It would be interesting to carry out a study with a larger sample size and to confirm the association between the T allele of the polymorphism rs235770 of the *BMP2* gene and the response to myopia control treatment with MiSight contact lenses, as well as studying its effect on different ethnic groups, ages, and gender.

Therefore, these findings will prove useful for future research in which detailed genetic mapping of the polymorphisms associated with myopia is performed to improve the strategies and interventions that are currently in place to slow the progression of myopia.

## 5. Conclusions

A pilot design study has been presented, which shows that the result of contact lens treatments for myopia control could vary depending on genetic variants.

The T allele, the one with the lowest frequency of the rs235770 polymorphism of the BMP2 gene, could have a significant effect on the response for the myopia control treatment.

Further studies with larger samples are needed to confirm the results of this pilot study.

## Figures and Tables

**Table 1:** Genes related to AL/CR ratio in children under 10 years old.

Gene	Locus	OMIM	SNP	Cambio	MAF	Beta Effect (SE) *	*p* *
*GJD2*	15q14	607058	rs524952	T > A	A: 0,471	0.0069 (0.0016)	10^−5^
*ZIC2*	13q32.3	603073	rs8000973	T > C	T: 0,475	0.0058 (0.0017)	10^−4^
*CHRNG*	2q37.1	100730	rs1881492	T > G	T: 0,224	0.0086 (0.0024)	10^−4^
*PRSS56*	2q37.1	613858	rs1656404	G > A	A: 0,220	0.0073 (0.0024)	0.002
*KCNQ5*	6q13	607357	rs7744813	A > C	C: 0,380	0.0050 (0.0017)	0.004
*SHISA6*	17p12	617327	rs2969180	G > A	A: 0,357	0.0035 (0.0016)	0.03
*KCNMA1*	10q22.3	600150	rs6480859	C > T	T: 0,362	0.0040 (0.0018)	0.02
*BMP2*	20p12.3	112261	rs235770	T > C	T: 0,379	0.0043 (0.0018)	0.02

OMIM: Online Mendelian Inheritance in Man; MAF: Minor allele frequency; AL/CR: axial length/corneal radius; T: thymine; A: adenine; G: guanine; C: cytosine; SNP: single-nucleotide polymorphism; SE: effect size; * according to Tideman et al. [7].

**Table 2:** Clinical characteristics of the study population.

Parameter	Responder (*n* = 15)	Nonresponder (*n* = 13)	*p*-Value *
Gender (*n*, %)	Female 8 (46.67) Male 7 (53.33)	Female 9 (69.23) Male 4 (30.77)	0.390
Age (mean ± SD, years)	17.47 ± 1.36	17.00 ± 1.63	0.416
AL (mean ± SD, mm)	Right: 24.57 ± 0.70 Left: 24.52 ± 0.75 Mean: 24.45 ± 0.71	Right: 25.23 ± 0.55 Left: 25.17 ± 0.53 Mean: 25.20 ± 0.53	0.011 0.014 0.011
SE (mean ± SD, D)	Right: −2.71 ± −1.41 Left: −2.55 ± 1.35 Mean: −2.62 ± 1.35	Right: −3.80 ± −1.48 Left: −4.16 ± 1.60 Mean: −3.98 ± 1.50	0.056 0.008 0.019
AL/CR (mean ± SD, mm)	Right: 0.58 ± 0.04 Left: 0.57 ± 0.04 Mean: 0.58 ± 0.01	Right: 0.60 ± 0.04 Left: 0.60 ± 0.04 Mean: 0.60 ± 0.01	0.071 0.059 0.064
Myopia degree (*n*, %)	Low myopia: 9 (63.3) Moderate myopia: 6 (36.7) High myopia: 0 (0)	Low myopia: 2 (19.2) Moderate myopia: 9 (65.4) High myopia: 2 (15.4)	0.001

AL: axial length; SE: spherical equivalent; * Qualitative variables: Chi-square test; Quantitative variables: Student’s *t*-test.

**Table 3:** Results of allelic association (adjusted for age, gender, and AL/CR).

Gene	SNP	Locus	Allele	MAF	Risk Allele	Risk Allele Frequency (%)		HWE *p*	*p*-Value	OR (95% CI)
						Res	N. Res			
*ZIC2*	rs8000973	13q32.3	T > C	0.446	T	12 (0.40)	13 (0.50)	0.72	0.61	1.44 (0.35–5.92)
*KCNQ5*	rs7744813	6q13	A > C	0.250	C	10 (0.33)	4 (0.15)	0.65	0.26	2.43 (0.50–11.75)
*GJD2*	rs524952	15q14	T > A	-	.	.	.	0.056	0.24	2.75 (0.48–15.57)
*CHRNG*	rs1881492	2q37.1	T > G	0.107	T	2 (0.07)	4 (0.15)	1	0.29	0.32 (0.04–2.81)
*PRSS56*	rs1656404	2q37.1	G > A	0.125	A	4 (0.13)	3 (0.12)	1	0.95	0.94 (0.12–7.38)
*BMP2*	rs235770	20p12.3	T > C	0.375	T	15 (0.5)	6 (0.23)	0.43	0.049	3.37 (1.079–10.886)

Res: Responders; N. Res: Nonresponders; MAF: Minor allele frequency; HWE: Hardy-Weinberg equilibrium.

**Table 4:** Results of genotypic association (adjusted for age, gender, and AL/CR).

Gene. (SNP)	Models of Heredity–Frequency (%)														
	Additive					Dominant					Recessive				
	Gn	Res *n* (%)	N. Res *n* (%)	*p*	OR (95% CI)	Gn	Res *n* (%)	N. Res *n* (%)	*p*	OR (95% CI)	Gn	Res *n* (%)	N. Res *n* (%)	*p*	OR (95% CI)
*ZIC2* (rs8000973)	C/C C/T T/T	5 (33.3) 8 (53.3) 2 (13.3)	4 (30.8) 5 (38.5) 4 (30.8)	0.48	1.00 4.31 (0.34–54.43) 2.32 (0.12–43.22)	C/C T/C + T/T	5 (33.3) 10 (66.7)	4 (30.8%) 9 (69.2%)	0.28	1.00 3.62 (0.32-40.86)	C/C + T/C T/T	13 (86.7) 2 (13.3)	9 (69.2) 4 (30.8)	0.81	1.00 0.77 (0.09–6.66)
*KCNQ5* (rs7744813)	A/A A/C C/C	6 (40.0) 8 (53.3) 1 (6.7)	9 (69.2) 4 (30.8) 0 (0)	0.45	1.00 2.08 (0.37–11.52) NA (0.00–NA)	A/A A/C + C/C	6 (40.0) 9 (60.0)	9 (69.2) 4 (30.8)	0.33	1.00 2.33 (0.43-12.66)	A/A + A/C C/C	14 (93.3) 1 (6.7)	13 (100.0) 0 (0.0)	0.34	1.00 NA (0.00–NA)
*GJD2* (rs524952)	A/A T/A T/T	2 (13.3) 10 (66.7) 3 (20.0)	2 (15.4) 10 (76.9) 1 (7.7)	0.37	1.00 1.38 (0.12–16.22) 10.21 (0.25-416.37)	A/A T/A + T/T	2 (13.3) 13 (86.7)	2 (15.4) 11 (84.6)	0.6	1.00 1.90 (0.18-20.46)	A/A+T/A T/T	12 (80.0) 3 (20.0)	12 (92.3) 1 (7.7)	0.16	1.00 7.93 (0.34–182.43)
*CHRNG* (rs1881492)	G/G G/T T/T	13 (86.7) 2 (13.3) 0 (0.0)	9 (69.2) 4 (30.8) 0 (0.0)	0.29	1.00 0.32 (0.04–2.81)	-	-	-	-	-	-	-	-	-	-
*PRSS56* (rs1656404)	G/G G/A A/A	11 (73.3) 4 (26.7) 0 (0.0)	10 (76.9) 3 (23.1) 0 (0.0)	0.95	1.00 0.94 (0.12–7.38)	-	-	-	-	-	-	-	-	-	-
*BMP2* (rs235770)	C/C C/T T/T	4 (26.7) 7 (46.7) 4 (26.7)	8 (61.5) 4 (30.8) 1 (7.7)	0.11	1.00 1.87 (0.26–13.63) 19.09 (0.84–434.64)	C/C C/T + T/T	4 (26.7) 11 (73.3)	8 (61.5) 5 (38.5)	0.15	1.00 3.58 (0.62–20.53)	C/C + C/T T/T	11 (73.3) 4 (26.7)	12 (92.3) 1 (7.7)	0.043	1.00 15.19 (0.72–319.94)

Gn: Genotype; Res: Responders; N. Res: Nonresponders; NA: No affected

**Table 5:** Results of allelic association (adjusted for age, gender, and SE).

Gene	SNP	Locus	Allele	MAF	Risk Allele	Risk Allele Frequency (%)		HWE *p*	*p*-Value	OR (95% CI)
						Res	N. Res			
*ZIC2*	rs8000973	13q32.3	T > C	0.446	T	12 (0.40)	13 (0.50)	0.72	0.78	0.82 (0.22–3.16)
*KCNQ5*	rs7744813	6q13	A > C	0.250	C	10 (0.33)	4 (0.15)	0.65	0.33	2.49 (0.38–16.10)
*GJD2*	rs524952	15q14	T > A	-	.	.	.	0.056	0.52	1.78 (0.29–10.81)
*CHRNG*	rs1881492	2q37.1	T > G	0.107	T	2 (0.07)	4 (0.15)	1	0.09	0.11 (0.01–1.73)
*PRSS56*	rs1656404	2q37.1	G > A	0.125	A	4 (0.13)	3 (0.12)	1	0.88	0.83 (0.08–9.09)
*BMP2*	rs235770	20p12.3	T > C	0.375	T	15 (0.5)	6 (0.23)	0.43	0.009	10.54 (1.00–111.38)

Res: Responders; N. Res: Nonresponders.

**Table 6:** Results of genotypic association (adjusted for age, gender, and SE).

Gene (SNP)	Models of Heredity–Frequency (%)														
	Additive					Dominant					Recessive				
	Gn	Res *n* (%)	N. Res *n* (%)	*p*	OR (95% CI)	Gn	Res *n* (%)	N. Res *n* (%)	*p*	OR (95% CI)	Gn	Res *n* (%)	N. Res *n* (%)	*p*	OR (95% CI)
*ZIC2* (rs8000973)	C/C C/T T/T	5 (33.3) 8 (53.3) 2 (13.3)	4 (30.8) 5 (38.5) 4 (30.8)	0.85	1.00 1.53 (0.09–25.16) 0.74 (0.05–11.31)	C/C T/C+ T/T	5 (33.3) 10 (66.7)	4 (30.8%) 9 (69.2%)	0.98	1.00 1.04 (0.09–12.06)	C/C + T/C T/T	13 (86.7) 2 (13.3)	9 (69.2) 4 (30.8)	0.63	1.00 0.58 (0.06–5.24)
*KCNQ5* (rs7744813)	A/A A/C C/C	6 (40.0) 8 (53.3) 1 (6.7)	9 (69.2) 4 (30.8) 0 (0.0)	0.61	1.00 2.42 (0.36–16.21) NA (0.00-NA)	A/A A/C + C/C	6 (40.0) 9 (60.0)	9 (69.2) 4 (30.8)	0.34	1.00 2.48 (0.37–16.54)	A/A + A/C C/C	14 (93.3) 1 (6.7)	13 (100.0) 0 (0.0)	0.72	1.00 NA (0.00-NA)
*GJD2* (rs524952)	A/A T/A T/T	2 (13.3) 10 (66.7) 3 (20.0)	2 (15.4) 10 (76.9) 1 (7.7)	0.66	1.00 0.94 (0.07–13.35) 4.28 (0.09-205.58)	A/A T/A + T/T	2 (13.3) 13 (86.7)	2 (15.4) 11 (84.6)	0.84	1.00 1.30 (0.10–16.37)	A/A + T/A T/T	12 (80.0) 3 (20.0)	12 (92.3) 1 (7.7)	0.36	1.00 4.45 (0.15–135.65)
*CHRNG* (rs1881492)	G/G G/T T/T	13 (86.7) 2 (13.3) 0 (0.0)	9 (69.2) 4 (30.8) 0 (0.0)	0.085	1.00 0.11 (0.01–1.73)	-	-	-	-	-	-	-	-	-	-
*PRSS56* (rs1656404)	G/G G/A A/A	11 (73.3) 4 (26.7) 0 (0.0)	10 (76.9) 3 (23.1) 0 (0.0)	0.88	1.00 0.83 (0.08–9.09)	-	-	-	-	-	-	-	-	-	-
*BMP2* (rs235770)	C/C C/T T/T	4 (26.7) 7 (46.7) 4 (26.7)	8 (61.5) 4 (30.8) 1 (7.7)	0.028	1.00 5.99 (0.31-116.13) 168.90 (1.08–NA)	C/C C/T + T/T	4 (26.7) 11 (73.3)	8 (61.5) 5 (38.5)	0.046	1.00 9.98 (0.78-127.69)	C/C + C/T T/T	11 (73.3) 4 (26.7)	12 (92.3) 1 (7.7)	0.019	1.00 1.26 (0.519–57.169)

Gn: Genotypic; Res: Responders; N. Res: Nonresponders.

**Table 7:** Linkage disequilibrium analysis.

Number of SNPs	Linkage Disequilibrium	*r*	*p*
SNP1–SNP6	rs235770–rs8000973	0.1735	0.1941
SNP2–SNP6	rs235770–rs7744813	0.0426	0.7496
SNP3–SNP6	rs235770–rs524952	0.1540	0.2491
SNP4–SNP6	rs235770–rs1881492	−0.2678	0.0451
SNP5–SNP6	rs235770–rs1656404	−0.2925	0.0286

**Table 8:** Haplotype analysis.

Haplotypes	Haplotype Frequency		OR (95% CI)	Haplotype Test
	Res	N. Res		*p*
rs235770–rs1656404				0.00011
GC	0.367	0.719	1.00	-
GT	0.500	0.165	22.99 (2.50–211.11)	0.008
AC	0.135	0.050	5.06 (0.54–47.23)	0.160

Res: Responders; N. Res: Nonresponders.
